# The role of auto-HSCT in extranodal natural killer/T cell lymphoma

**DOI:** 10.1515/med-2024-1024

**Published:** 2024-10-03

**Authors:** Yin-yin Peng, Xin Wang, Lin Liu

**Affiliations:** Department of Hematology Medicine, The First Affiliated Hospital of Chongqing Medical University, Chongqing, 400016, China

**Keywords:** extranodal NK/T-cell lymphoma, chemotherapy, autologous, hematopoietic stem cell transplantation

## Abstract

**Objectives:**

Autologous hematopoietic stem cell transplantation (auto-HSCT) is considered optional consolidation therapy especially for relapsed/refractory extranodal NK/T-cell lymphoma (ENKL), but its applications to newly diagnosed advanced-stage ENKL is currently limited.

**Methods:**

We collected 51 cases of newly diagnosed advanced-stage ENKL patients, including 26 with auto-HSCT and 25 with chemotherapy rather than HSCT, from our hospital between 2014/01 and 2023/12. We summarized the patients’ characteristics, conducted survival analysis of the 51 cases, and analyzed the potential benefits of auto-HSCT to ENKL patients.

**Results:**

It shows that after a median follow-up time of 39 months, the estimated 5-year overall survival (OS) of the 51 newly diagnosed advanced-stage ENKL patients is 73.4%, and their estimated 5-year progression-free survival (PFS) is 73.4%. For patients receiving auto-HSCT, the 5-year OS (91.7%) and PFS (91.0%) are significantly different from those of patients receiving chemotherapy without HSCT (OS 53.3%, PFS 54.5%) (*p* < 0.05). Univariate and multivariate analysis results suggest that only the l-asparaginase usage in chemotherapy showed significant impact on the OS, and none of concerned factors showed significant impact on the PFS.

**Conclusions:**

Auto-HSCT is indeed an option to newly diagnosed advanced-stage ENKL, but further studies are still required for more strict disease management.

## 1 Introduction

Extranodal NK/T-cell lymphoma (ENKL) is rarely diagnosed in Western countries but relatively more common in East Asian countries. It is closely associated with Epstein-Barr virus (EBV). Among newly diagnosed advanced and limited stage of ENKLs, the limited-stage ENKL responds relatively much better to radiotherapy or concurrent radiation and chemotherapy. Regimes containing l-asparaginase are effective to limited-stage ENKL, but are not that satisfactory to advanced-stage ENKL [1]. Studies show that the complete remission (CR) rate of advanced-stage patients with l-asparaginase, etoposide, and dexamethasone (AspaMetDex) regimen was 30%, and the 5-year survival rate of those patients with the l-asparaginase, vincristine, and dexamethasone regimen was only 25% [2,3]. As advanced-stage ENKLs are highly progressive and sometimes multi-drug resistant, there is so far no standard management for advanced-stage ENKLs.

In most clinical management guidelines and expert consensuses, autologous hematopoietic stem cell transplantation (auto-HSCT) is considered as an optional consolidation therapy for newly diagnosed advanced-stage ENKL. However in clinic, the rules for choice of auto-HSCT to high-risk ENKL patients are still unclear. Previous papers about auto-HSCT mostly talked not specifically about ENKL, but generally about peripheral T-cell lymphoma, including many other subtypes like anaplastic large-cell lymphoma, angioimmunoblastic T-cell lymphoma, hepatosplenic T-cell lymphoma, subcutaneous panniculitis-like T-cell lymphoma, enteropathy-associated T-cell lymphoma, cutaneous T-cell lymphoma, and aggressive NK-cell leukemia [4–8].

In this study, in order to provide more insights into the rules for choice of auto-HSCT, we summarized and analyzed the clinical cases of newly diagnosed advanced-stage ENKLs from our hospital (the First Affiliated Hospital of Chongqing Medical University) between 2014/01 and 2023/12. From our hospital, 51 newly diagnosed advanced-stage ENKL patients were included in this analysis, of which 26 underwent auto-HSCT, the other 25 received chemotherapy without HSCT. We summarized the characteristics of all 51 newly diagnosed advanced-stage ENKL patients and evaluated potential benefits of these characteristics to ENKL patients. We hope to provide insights into the clinical practice of auto-HSCT to ENKL patients.

## 2 Materials and methods

### 2.1 Patients

From the department of hematology at our hospital, between 2014/01 and 2023/12, we retrospectively analyzed the clinical data of newly diagnosed patients with advanced-stage ENKL, and compared the data of patients with auto-HSCT to that of patients without auto-HSCT. As auto-HSCT is usually performed in patients with age below 60 years, patients older than 60 years were excluded during data collection.

Totally 51 newly diagnosed advanced-stage ENKL patients were collected retrospectively, of which 26 received auto-HSCT, while the other 25 received chemotherapy without HSCT. The choice of auto-HSCT was first recommended to but finally determined by the patients themselves. For young (age <60 years) patients, we would like to recommend auto-HSCT, but still the final decision is up to the patients themselves. All patients were diagnosed by lymph node biopsy and were confirmed by pathology, according to the criteria of the World Health Organization for the classification of lymphoid tissue tumors in 2016. Following the guidelines of American National Comprehensive Cancer Network [9], the prognostic indicators of natural killer cell lymphoma containing Epstein-Barr virus DNA (PINK-E) were selected for prognostic evaluation and risk stratification. Disease stages of the patients were classified according to a modification of the Ann Arbor system.

### 2.2 Transplantation

About 24/26 patients received chemotherapy without radiotherapeutic initial treatment before transplant. Two of them had external radiotherapy for nasopharynx lymphomas. They all received either l-asparaginase-containing chemotherapy or l-asparaginase-absent chemotherapy. After that, they received peripheral blood hematopoietic stem cells (PBSC) transplantation. Granulocyte colony stimulating factor 10 mg/kg per day was applied to patients for the mobilization of PBSC. Their conditioning regimens were myeloablative, including lomustine, etoposide, cytarabine, and cyclophosphamide (CEAC) for eight auto-HSCT patients; carmustine, etoposide, cytarabine, and melphalan (BEAM) for 16 auto-HSCT patient; idarubicin, etoposide, cytarabine, and cyclophosphamide (IEAC) for two auto-HSCT patients (details in Table 1). All other 25 patients received chemotherapy containing l-asparaginase without HSCT (details in Table 2).

**Table 1:** Clinical data of 26 newly diagnosed advanced-stage ENKL patients treated with auto-HSCT in our hospital

Patient no.	Gender	Age	Stage	Bone marrow infiltration	LDH	PINK-E score	Chemotherapy before auto-HSCT	l-asparaginase-containing chemotherapy before auto-HSCT	Disease status at auto-HSCT	Radiotherapy	Conditioning regimens	OS (month)	PFS (month)	Alive
1	Male	36	III	No	N	3	3	Yes	PR	No	CEAC	73	73	Yes
2*	Female	25	IV	No	E	3	5	No	PR	No	CEAC	15	10	No
3	Male	42	IV	No	E	3	5	Yes	CR	No	CEAC	83	83	Yes
4**	Male	46	IV	No	N	3	6	No	PR	No	CEAC	17	17	No
5#	Female	42	IV	No	E	4	2	Yes	CR	No	CEAC	83	23	Yes
6	Male	26	III	No	N	3	4	Yes	CR	No	CEAC	115	115	Yes
7	Male	19	IV	No	N	4	4	Yes	CR	No	CEAC	85	85	Yes
8	Male	21	III	No	N	3	4	Yes	CR	No	BEAM	58	58	Yes
9	Female	41	III	No	N	3	4	Yes	CR	No	IEAC	65	65	Yes
10	Female	24	IV	No	N	3	5	Yes	CR	No	CEAC	60	60	Yes
11	Female	54	IV	Yes	N	3	5	Yes	CR	No	IEAC	59	59	Yes
12	Female	25	IV	No	E	3	6	Yes	CR	No	BEAM	56	56	Yes
13	Female	52	IV	No	N	3	3	Yes	CR	No	BEAM	55	55	Yes
14	Female	33	IV	Yes	E	3	5	Yes	CR	No	BEAM	57	57	Yes
15	Female	34	IV	No	N	3	5	Yes	CR	No	BEAM	45	45	Yes
16	Female	35	IV	Yes	E	3	6	Yes	CR	No	BEAM	27	27	Yes
17	Male	31	IV	No	N	3	4	Yes	PR	No	BEAM	25	25	Yes
18	Female	36	IV	No	N	3	5	Yes	CR	No	BEAM	23	23	Yes
19	Female	56	IV	No	N	4	3	Yes	CR	No	BEAM	22	22	Yes
20	Male	35	IV	No	E	3	5	Yes	CR	No	BEAM	27	27	Yes
21	Male	42	IV	No	N	3	6	Yes	CR	No	BEAM	30	30	Yes
22	Male	38	IV	No	N	3	6	Yes	PR	No	BEAM	32	32	Yes
23	Male	37	IV	No	E	3	4	Yes	CR	No	BEAM	56	56	yes
24	Male	30	III	No	E	3	5	Yes	CR	Yes	BEAM	9	9	Yes
25	Male	59	IV	No	E	3	5	Yes	CR	No	BEAM	8	8	Yes
26	Male	34	IV	No	N	3	5	Yes	CR	Yes	BEAM	27	12	Yes

Abbreviations: ENKL, extranodal NK/T-cell lymphoma; LDH, lactate dehydrogenase; PINK-E, prognostic indicators of natural killer cell lymphoma containing Epstein-Barr virus DNA; auto-HSCT, autologous hematopoietic stem cell transplantation; allo-HSCT, allogeneic hematopoietic stem cell transplantation; OS, overall survival; PFS, progression-free survival; E, elevated; N, normal; CR, complete remission; PR, partial remission; CEAC, lomustine, etoposide, cytarabine, and cyclophosphamide; BEAM, carmustine, etoposide, cytarabine, and melphalan; IEAC, idarubicin, etoposide, cytarabine, and cyclophosphamide.

*The patient replased in 10 months after being diagnosed, then received chemotherapy of GLIDE (gemcitabine, pegaspargase, ifosfamide, dexamethasone, and etoposide)+chidamide, but died 15 months after being diagnosed because of disease progression.

**The patient remained CR, but died of severe pneumonia 17 month after being diagnosed.

#The patient replased in 23 months after auto-HSCT, then received four cycles of chemotherapy of P-GEMOX, maintained with chidamide without any disease progression for 76 month after being diagnosed.

**Table 2:** Clinical data of 25 newly diagnosed advanced-stage ENKL patients treated with chemotherapy without HSCT in our hospital

Patient no.	Gender	Age	Stage	Bone marrow infiltration	LDH	PINK-E score	l-asparaginase-containing chemotherapy	Chemotherapy cycle	Radiotherapy	OS (month)	PFS (month)	Alive
1	Male	53	III	No	N	2	Yes	8	No	46	40	No
2	Male	47	III	No	E	3	Yes	8	No	54	54	Yes
3	Male	14	IV	Yes	E	3	Yes	8	No	54	54	Yes
4	Male	51	IV	No	E	3	Yes	9	No	15	12	No
5	Male	45	IV	No	E	3	Yes	6	No	10	7	No
6	Male	53	III	No	E	3	Yes	8	Yes	109	109	Yes
7	Male	45	III	No	N	2	Yes	6	Yes	122	122	Yes
8	Female	39	IV	No	N	4	Yes	4	No	4	4	No
9	Male	52	IV	No	N	3	Yes	8	Yes	64	64	Yes
10	Female	49	IV	Yes	E	4	Yes	1	No	1	1	No
11	Male	55	IV	Yes	E	4	Yes	8	No	61	61	Yes
12	Male	42	IV	No	N	3	Yes	6	No	8	8	No
13	Male	51	III	Yes	E	3	Yes	3	No	3	3	No
14	Female	27	IV	No	E	3	Yes	3	No	36	36	Yes
15	Female	47	IV	No	E	3	Yes	6	No	5	5	No
16	Male	55	IV	Yes	N	3	Yes	8	No	33	33	Yes
17	Female	58	IV	Yes	N	3	Yes	1	No	1	1	No
18	Male	22	IV	Yes	E	3	Yes	5	No	34	34	Yes
19	Female	58	IV	Yes	E	4	Yes	4	No	21	21	Yes
20	Female	34	IV	Yes	N	3	Yes	5	No	12	12	Yes
21	Male	40	IV	No	E	3	Yes	6	No	9	9	Yes
22	Male	57	IV	No	E	3	Yes	6	No	16	16	Yes
23	Male	22	IV	No	E	3	Yes	3	No	14	11	No
24	Female	16	III	No	N	2	Yes	4	No	13	13	Yes
25	Male	59	IV	No	N	3	Yes	6	Yes	18	18	Yes

Abbreviations: ENKL, extranodal NK/T-cell lymphoma; LDH, lactate dehydrogenase; PINK-E, prognostic indicators of natural killer cell lymphoma containing Epstein-Barr virus DNA; OS, overall survival; PFS, progression-free survival; E, elevated; N, normal.

### 2.3 Statistical methods

Overall evaluation of the patients was performed based on the following information: medical record review, physical examination, blood chemistry, computed tomography (CT) imaging (total body positron emission tomography/CT or CT of the head, neck, chest, and abdomen), ultrasound imaging of systemic superficial lymph nodes, and bone marrow aspiration.

We not only summarized the characteristics of the patients during the treatment in our hospital, but also performed survival analysis to those cases. The overall survival (OS) is the time from diagnosis to death, or to the last follow-up time which was set on 2024/04/30 in this study. The progression-free survival (PFS) is the time from diagnosis to relapse, disease progression, death, or to the last follow-up time. The OS and PFS were estimated using Kaplan–Meier method in IBM SPSS v23.0, survival comparisons were made using the log-rank test. The impacts of factors to the OS and PFS were evaluated using Kaplan–Meier regression for univariate analysis and using Cox regression for multivariate analysis. The status variables for OS and PFS were survival and progression, respectively. And a *p* value (two-tailed) <0.05 was considered statistically significant.


**Ethical approval:** The study was approved by the ethics committee of the First Affiliated Hospital of Chongqing Medical University (number: 2021-504).
**Informed consent:** This study was a retrospective study, HSCT treatment was not administered for the purpose of research, consent to participate for the purpose of study was not required; however, it is a routine procedure of our hospital that patients must sign informed consent before any treatment. Obtaining consent is also definitely required before transplantation. Consent to publish was obtained after the study when we were preparing the manuscript, following the suggestion of the director of our department according to the regulations of our department.

## 3 Results

### 3.1 Patient characteristics

Of the 51 newly diagnosed advanced-stage ENKL patients, 26 received auto-HSCT. They included 14 males and 12 females with median age 37 years (range 19–59) (Table 1). They were all in good physical conditions with the Eastern Cooperative Oncology Group score 0–1. At diagnosis, their serum EBV-DNAs value was high, their tumor proliferation indices (Ki-67) were ≥40%, and their PINK-E indicated high-risk lymphomas. As the first-line chemotherapy, l-asparaginase-containing regimens were applied to 24 of them (92.3%) for 2–6 cycles, and l-asparaginase-absent regimens were applied to the other two (7.7%) for 5–6 cycles, with the overall median cycle 5. Before auto-HSCT, 80.8% (21/26) of them got CR, while 19.2% (5/26) were in partial remission (PR) condition. After auto-HSCT, their conditioning regimens included BEAM (applied to 16 patients), CEAC (to 8), and IEAC (to 2).

The other 25 newly diagnosed advanced-stage ENKL patients received chemotherapy without HSCT. All of them received chemotherapy containing l-asparaginase for 1–9 cycles (median cycle 6). They were 17 males and 8 females with median age 44 years (range 14–59) (Table 2).

### 3.2 Outcomes

The PBSCs of all 26 patients showed complete engraftment within 3 weeks without any engraftment failure. Of the 26 patients with auto-HSCT, their median follow-up time is 47 months (range: 8–115 months). After auto-HSCT, two patients died of disease progression and severe pneumonia in 15 and 17 months, respectively, other 24 achieved CR condition without disease progression and survived for at least 8 months after auto-HSCT. The other of the 24 (No. 5 patient) auto-HSCT patients relapsed 23 months later after transplantation. He then received four cycles of chemotherapy of gemcitabine, oxaliplatin, and pegaspargase (P-GEMOX), and was maintained with chidamide without any disease progression for at least 83 months after auto-HSCT (details in Table 1).

### 3.3 Survival analysis

**Figure 1:**
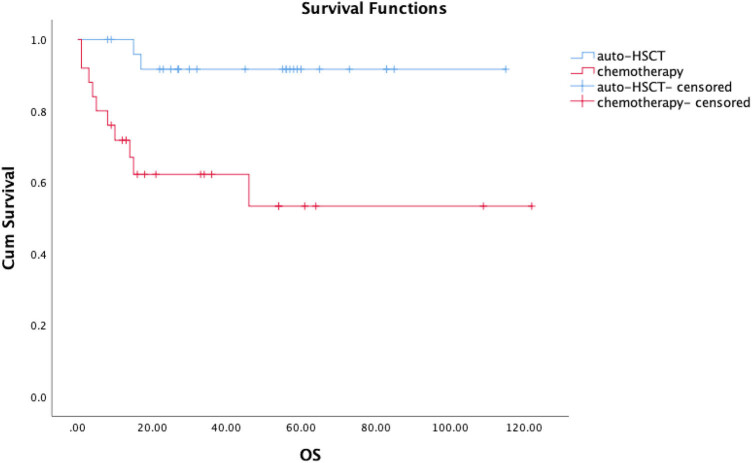
Kaplan-Meier curves of OS for newly diagnosed advanced-stage ENKL patients, patients received auto-HSCT vs chemotherapy in our hospital.

**Figure 2:**
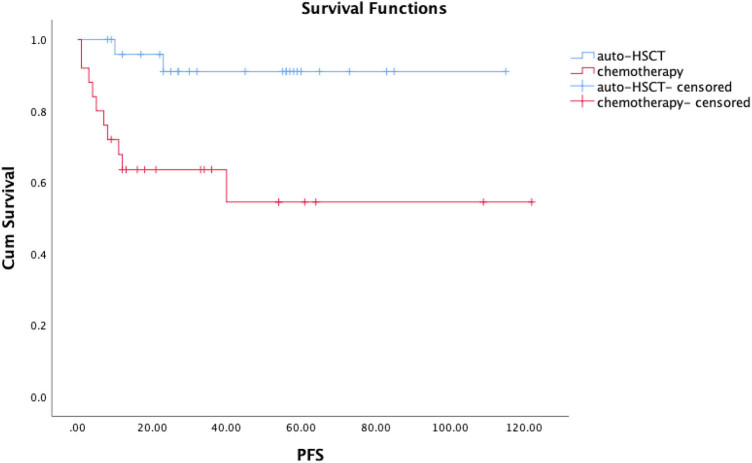
Kaplan-Meier curves of PFS for newly diagnosed advanced-stage ENKL patients, patients received auto-HSCT vs chemotherapy in our hospital.

After the median follow-up time (39 months), the estimated 5-year OS of the total 51 newly diagnosed advanced-stage ENKL patients is 73.4%, and their estimated 5-year PFS is 73.4%. For the patients with auto-HSCT, after the median follow-up time (47 months), the estimated 5-year OS is 91.7% (Figure 1) and the estimated 5-year PFS is 91.0% (Figure 2). After auto-HSCT, two patients relapsed but only one died of disease progression while the other got CR again after salvage chemotherapy. However, the 5-year OS in patients who received chemotherapy without HSCT is 53.3% (Figure 1), and the 5-year PFS is 54.5% (Figure 2), showing significant difference from those of auto-HSCT patients (*p* = 0.003 for OS, *p* = 0.004 for PFS).

We then conducted further regression analysis on potential impact factors to the OS and PFS for the total 51 patients. The focused impact factors we selected included sex, age (grouped into “>50 years” and “<50 years”), stage (“III” or “IV”), bone marrow infiltration (“presence” or “absence”), LDH (“elevated” or “normal”), PINK-E score, l-asparaginase regimens used in chemotherapy (“yes” or “not”), radiotherapy (“received” or “not received”). Results show that only the l-asparaginase usage in chemotherapy showed significance in both the univariate analysis (*p* = 0.024) and the multivariate analysis (*p* = 0.027), and none of these factors showed significant impact on the PFS.

## 4 Discussion

ENKL is a relatively rare non-Hodgkin lymphoma with poor prognosis. Many previous studies have reported poor survival outcome in patients with ENKL. For ENKL treatment, auto-HSCT is typically used either for consolidation of first complete remission in advanced-stage ENKL with high PINK-E, or for the achievement of CR after relapse [10]. However, the roles of auto-HSCT in newly diagnosed advanced-stage ENKLs still remain unclear [10].

Thus, we conducted this analysis of outcomes of patients with newly diagnosed advanced-stage ENKL. In the department of hematology of the First Affiliated Hospital of Chongqing Medical University, between 2014/01 and 2023/12, we had treated over 100 ENKL patients, but most of them were early stage, only 51 of them were newly diagnosed with advanced-stage and high risk ENKL.

The estimated 5-year OS of the total 51 newly diagnosed advanced-stage ENKL patients is 73.4% and median survival of these patients is 39 months. Compared with previous studies, our result of 5-year OS is higher but still close to that reported in literature which ranges from 20 to –72% [11–16], and our result of median survival is consistent with that reported previously ranging from 13 to –67 months [11–16]. In this study, the estimated 5-year PFS is 73.4% which is higher than that in literature ranging from 22.6 to 46.8% [17–22]. The reason for the differences in the 5-year OS and PFS might be not only due to the limit of the case number and follow-up time of the cases we collected, but also more importantly, due to the high 5-year OS and PFS of the 26 auto-HSCT patients, which largely contribute to the overall 5-year OS and PFS of the total 51 patients.

Among them, 26 received auto-HSCT, while the other 25 received chemotherapy without HSCT. According to the analysis of cases from our hospital, the estimated 5-year OS and PFS for the 26 patients with auto-HSCT is 91.7 and 91.0%, respectively, which are much higher than those reported in literature. Our data showed that HSCT can significantly improve the OS and PFS compared to those patients who received chemotherapy without HSCT (*p* < 0.05). This finding is accordant to previous studies showing that patients with high-dose chemotherapy and auto-HSCT tend to have longer survival time than non-HSCT patients [23–26]. So auto-HSCT is indeed an option for advanced-stage ENKL, and is indeed beneficial for survival.

Some studies have reported that, for ENKL patients who received HSCT, the CR condition before transplantation might significantly improve the OS and PFS [6,10,23,27–29]. Moreover, chemotherapy of l-asparaginase prior to HSCT significantly improved the OS and PFS too [6]. As for the cases from our hospital, since the patients were young and newly diagnosed with ENKL, they were not relapsed/refractory, and most of them (24/26) received l-asparaginase containing chemotherapy and attained CR/PR without any stable disease or progressive disease, besides the follow-up time is short; therefore, the OS and PFS for those patients who received auto-HSCT from our hospital were high.

It was also reported that, pretreatment PINK-E scores and chemotherapy regimen were strongly associated with OS and PFS [30,31]. But we find that only the l-asparaginase usage showed significant impact on only the OS rather than PFS. More cases need be included for further study in the future.

We searched in PubMed, Medline, and Embase for publications about auto-HSCT in ENKL patients. Most of the papers only reported very limited number of cases often even without randomized controlled trials. At present, the largest number of cases reported of HSCT for ENKL patients is a multicenter retrospective study on consecutively collected patient data from the Japanese nation-wide transplant registry database [32]. In that study, patients with all subtypes of lymphoma who received first auto-HSCT from 1980/01 to 2018/12 were analyzed, including 195 ENKL patients with auto-HSCT. However, the paper reported that the 5-year OS from event-free survival (EFS) was 89.9 and 93.8% for 24 and 60 months, respectively, in auto-HSCT ENKL patients, which implies that the relapse and death rate may decrease in the later period of HSCT, and the EFS status at 24 or 60 months could be an early end point after HSCT.

ENKL patients especially at advanced-stage usually have poor prognosis that nearly half of newly diagnosed patients experience continuous disease progression. It implies that the HSCT, as a front-line consolidation treatment, decreases the relapse rate, and helps improve outcomes in advanced-stage ENKL patients [33]. However, HSCT tends to sacrifice the treatment-related mortality (TRM) for long-term remission [33]. According to published literature, for patients after auto-HSCT, the TRM is mostly close to 0 [6,27,34], but it was once also reported in a study that the TRM was 14.3% [28]. So auto-HSCT is likely to be promising, but requires more medical experience and more strict disease management.

Allogeneic hematopoietic stem cell transplantation (allo-HSCT) is a potential curative treatment to high-risk lymphomas, owing to its associated graft-versus-lymphoma effect [1]. Asian experts propose that upfront allo-HSCT is beneficial when used in high-risk ENKL patients [35,36]. As those papers and guidelines may be subjected to personal subjective experiences and judgments, further studies are still needed to contribute to this topic.

## 5 Conclusion and limitation

In this study, we reported 51 advanced-stage of newly diagnosed ENKL patients collected from our hospital in recent 10 years, of which 26 received auto-HSCT, while the other 25 received chemotherapy without HSCT. Due to the rarity of ENKL and the difficulty in conducting randomized controlled trials, the optimal treatment regimen of ENKL is still undetermined. But at least we can confirm that auto-HSCT is able to benefit the OS and PFS for the advanced-stage of newly diagnosed ENKL patients, although at present the choice of auto-HSCT is mainly based upon subjective expertise, bringing heavy selection bias. Although the exact role of auto-HSCT is still unclear, they are indeed potential options to advanced stage ENKL. Novel treatments such as allo-HSCT [22], anti-CD30 antibody [37], programmed death protein ligand 1 [38], and histone deacetylase (HDAC) inhibitors [39] have been reported as feasible choices for advanced stage ENKLs.

## Abbreviations

allo-HSCT: allogeneic hematopoietic stem cell transplantation
auto-HSCT: autologous hematopoietic stem cell transplantation
BEAM: carmustine, etoposide, cytarabine, melphalan
CEAC: lomustine, etoposide, cytarabine, cyclophosphamide
CR: complete remission
EBV: Epstein-Barr virus
ECOG: Eastern Cooperative Oncology Group
IEAC: idarubicin, etoposide, cytarabine, cyclophosphamide
EFS: event-free survival
ENKL: extranodal NK/T-cell lymphoma
G-CSF: granulocyte colony stimulating factor
HDAC: histone deacetylase
NCCN: National Comprehensive Cancer Network
NHL: non-Hodgkin lymphoma
OS: overall survival
PBSC: peripheral blood hematopoietic stem cell
PD: progressive disease
PD-L1: programmed death protein ligand 1
PET/CT: positron emission tomography/computed tomography
PFS: progression-free survival
P-GEMOX: gemcitabine, oxaliplatin, pegaspargase
PINK-E: prognostic indicators of natural killer cell lymphoma containing Epstein-Barr virus DNA
PR: partial remission
SD: stable disease
TRM: treatment-related mortality
WHO: World Health Organization
